# Yield of FDG PET/CT for Defining the Extent of Disease in Patients with Kaposi Sarcoma

**DOI:** 10.3390/cancers14092189

**Published:** 2022-04-27

**Authors:** Louise Pesqué, Julie Delyon, Coralie Lheure, Barouyr Baroudjian, Maxime Battistella, Pascal Merlet, Céleste Lebbé, Laetitia Vercellino

**Affiliations:** 1 Nuclear Medicine Department, Saint Louis University Hospital, Assistance-Publique Hôpitaux de Paris, 1 Avenue Claude Vellefaux, 75010 Paris, France; louise.pesque@gmail.com (L.P.); pascal.merlet@aphp.fr (P.M.); 2 Department of Dermatology, Saint Louis University Hospital, Assistance-Publique Hôpitaux de Paris, 1 Avenue Claude Vellefaux, 75010 Paris, France; julie.delyon@aphp.fr (J.D.); barouyr.baroudjian@aphp.fr (B.B.); celeste.lebbe@aphp.fr (C.L.); 3 INSERM HIPI Team 1, U976, Saint Louis University Hospital, 1 Avenue Claude Vellefaux, 75010 Paris, France; maxime.battistella@aphp.fr; 4 Université de Paris Cité, 75006 Paris, France; coralie.lheure@aphp.fr; 5 Department of Dermatology, Cochin University Hospital, 27, Rue du Faubourg-Saint-Jacques, 75014 Paris, France; 6 Department of Pathology, Saint Louis University Hospital, Assistance-Publique Hôpitaux de Paris, 1 Avenue Claude Vellefaux, 75010 Paris, France; 7 Université de Paris, INSERM, UMR_S942 MASCOT, 75006 Paris, France

**Keywords:** F-18-Fluorodeoxyglucose, positron emission tomography/computed tomography, Kaposi sarcoma, disease staging, diagnostic accuracy

## Abstract

**Simple Summary:**

The potential role of positron emission tomography/computed tomography with fluorodeoxyglucose (FDG PET/CT) for assessing the extent of Kaposi sarcoma is not well studied. We analyzed FDG PET/CTs performed on 75 patients referred to our department for Kaposi sarcoma staging or restaging. FDG PET/CTs detected most lymph nodes, bone, and muscle lesions, whereas digestive and mucous lesions could be missed. Most cutaneous lesions can be identified when whole-body FDG PET/CT (including lower limbs) is performed. Thus, a true whole-body FDG PET/CT can be recommended for staging purposes in patients with active Kaposi sarcoma and, if positive, be useful for therapeutic evaluation and follow-up.

**Abstract:**

Background: Positron emission tomography/computed tomography with fluorodeoxyglucose (F-18) (FDG PET/CT) is increasingly used in Kaposi sarcoma (KS), but its value has not been assessed. Objectives: In this study, we aimed to evaluate the diagnostic accuracy of FDG PET/CT to define the extent of disease in KS. Methods: Consecutive patients with KS referred to our department for FDG PET/CT were included. The diagnostic accuracy of FDG PET/CT for cutaneous and extra-cutaneous KS staging was assessed on a per lesion basis compared to staging obtained from clinical examination, standard imaging, endoscopy, histological analyses, and follow-up. Results: From 2007 to 2017, 75 patients with FDG PET/CT were analyzed. The sensitivity and specificity of FDG PET/CT for the overall detection of KS lesions were 71 and 98%, respectively. Sensitivity and specificity were 100 and 85% for lymph nodes, 87 and 98% for bone, 87 and 100% for lungs, and 100 and 100% for muscle involvement, whereas sensitivity was only 17% to detect KS digestive involvement. The sensitivity of the diagnostic for KS cutaneous involvement increased from 73 to 88% when using a whole-body examination. Conclusion: FDG PET/CT showed good sensitivity and specificity for KS staging (digestive involvement excepted) and could be used for staging patients with active KS.

## 1. Introduction

Kaposi sarcoma (KS) is a chronic proliferative disease developed at the expense of vascular endothelium with a dual component of vascular and fibroblastic cells associated with Human Herpes Virus 8 (HHV8) infection [1].

Four types of KS are described: classic (sporadic or Mediterranean), endemic (African), epidemic (or HIV-related), and iatrogenic (induced by immunosuppressive drugs) [1,2,3,4]. It mostly involves the skin (especially in distal limbs), but the KS extension to deeper structures such as bone and joints can be destructive and lead to severe functional impairment [5]. Musculoskeletal involvement is mostly seen in endemic and HIV-related KS [6]. Visceral lesions are rare in immunocompetent patients but can be rapidly progressive and life-threatening in immunocompromised patients [3,5]. Gastrointestinal involvement is the most common visceral site, involved in 40–51% of untreated HIV-related KS, and can induce severe gastrointestinal bleeding [4,7]. Pulmonary involvement can also be observed, especially in iatrogenic and epidemic KS [8,9].

Therapeutic strategies for KS are multiple, depending on KS type, severity, and extension to visceral organs. Therapeutic options are based on local (for instance, surgery, radiotherapy, alitretinoin) or systemic therapies (interferon, chemotherapy, and more recently, PD1 blockade) [1,2,10,11,12,13], in addition to the adaptation of immunosuppression and antiretroviral therapy for patients with iatrogenic or HIV-related KS. Thus, assessing the KS extension is of utmost importance to guide therapeutic choice. Recent guidelines limited indications of imaging work-up to patients with progressive disease or presenting symptoms suggesting visceral involvement [14]. The current staging work-up is based on a multimodal strategy, including computed tomography (CT), magnetic resonance imaging (MRI), upper and lower digestive endoscopy, and/or bronchial endoscopy [14,15,16]. However, multiple diagnostic procedures may be time-consuming, and endoscopies are invasive, thus raising interest in a single whole-body procedure.

The role of hybrid positron emission tomography/computed tomography using F-18-Fluorodeoxyglucose (FDG PET/CT) is well-established in cancer imaging. FDG PET/CT can provide a whole-body assessment of a proliferative or inflammatory disease, delivering metabolic and morphologic information concomitantly. Some cases of patients with KS successfully assessed with FDG PET/CT were reported, suggesting that it may be a useful tool to assess KS extension or treatment response [17,18,19,20,21,22]. However, no study measuring the efficacy of FDG PET/CT in KS has been reported to date. In order to assess the diagnostic accuracy of FDG PET/CT for KS staging, we conducted a large retrospective study on patients who had an FDG PET/CT examination for KS.

## 2. Materials and Methods

### 2.1. Patient Population

The inclusion criteria were patients diagnosed with any type of KS in the Dermatology department of Saint-Louis Hospital, Paris, France, who had an FDG PET/CT for KS and a minimum follow-up 6 months after FDG PET/CT. If a patient had several FDG PET/CTs, the first one was considered for this study. Clinical data, KS characteristics, and outcomes were collected from medical files.

This retrospective study was approved by the institutional review board CEERB Paris Nord (IRB number: 00006477; project number: 2018-012).

### 2.2. Image Acquisition and Reconstruction

A standardized imaging protocol was used [23]. Briefly, well-hydrated patients fasted for 6 h before undergoing PET/CT. The patients’ blood glucose level had to be <7 mmol/L. A median activity of 4.8 +/− 0.9 MBq/kg (0.129 +/− 0.024 mCi) of body weight for ^18^F-FDG was administered intravenously 1 h before imaging. CT images were obtained from the skull base to the knee/mid-thigh or the whole body (no oral or intravenous contrast agent). Most FDG PET/CTs were performed in patients with a potential risk of extra-cutaneous involvement, assessment of lymph nodes, and visceral involvement; therefore, systematic full-body FDG PET/CT was not always performed. However, if a patient reported a symptomatology of lower limb pain or history of KS bone involvement, a whole-body PET was performed to assess muscles and bones. The CT transmission map was used for attenuation correction.

### 2.3. Image Interpretation

FDG PET/CT images were retrospectively and independently analyzed by two experienced nuclear physicians (LP and LV) blinded to the clinical history and results of previous imaging. PET (attenuation-corrected and non-corrected images), CT, and fused images were analyzed.

Any abnormal focus of increased FDG uptake was classified as KS-related or KS-unrelated based on its intensity of uptake and pattern. Each lesion considered KS-related was assigned to skin or an extra-cutaneous site: mucous (oral or genital), bones, lymph nodes, lung, digestive tract, muscle, ENT (ear, nose, and throat) area or liver, resulting in nine sites (including the skin) systematically analyzed per patient. SUV_max_ was measured for all identified lesions.

An FDG PET/CT examination with at least one lesion considered non-physiological and related to KS was defined as positive.

An FDG PET/CT was considered normal (or negative) if no lesion was detected or if all findings were considered benign or not related to KS.

### 2.4. Standard KS Staging and Determination of the Diagnostic Accuracy of FDG PET/CT

The determination of KS disease sites were defined by KS extension to cutaneous and extracutaneous sites assessed by clinical examination, available standard imaging (CT and MRI), endoscopy, and/or pathology [11], and in all cases from follow-up data, thus defining the gold standard for staging (“standard staging”). Follow-up data included results from physical examination and standard imaging if performed.

FDG PET/CT results were compared with “standard staging” at the time of the FDG PET/CT examination. FDG PET/CT staging (PET staging) was classified as true positive (TP), true negative (TN), false positive (FP), or false negative (FN). Per site analysis: the site was considered TP if at least one uptake was identified in the studied organ and confirmed by standard staging. The site was considered TN if no uptake was considered KS-related in the corresponding site, which was confirmed by standard staging. The site was considered FP if the site involvement was not confirmed by standard staging. The site was considered FN if no uptake in the organ was observed while being diagnosed as involved by standard staging. Sensitivity, specificity, positive and negative predictive values, and the diagnostic accuracy (DA) of FDG PET/CT were calculated globally for all involved sites in the whole population and the subgroup with whole-body FDG PET/CT for cutaneous involvement and extra-cutaneous involvement. If all sites were considered TP per patient (or per examination analysis), the examination was considered TP. If all sites were considered TN, the examination was considered TN. If there were FN and TN lesions, the examination was considered FN. If there was an FP lesion with an FN or TN lesion, the patient was considered FP. Finally, if there were coexisting TP lesions with either TN, FP, or FN lesions, the examination was considered TP.

## 3. Results

### 3.1. Characteristics of Patients

From May 2007 to March 2017, 75 consecutive patients with an FDG-PET/CT for KS were included. The patients’ characteristics are summarized in Table 1.

The median age at KS diagnosis was 56 years. The median age at the time of the PET/CT examination was 65 years. The median time from KS diagnosis was 9 years. Twenty-eight patients (37%) had iatrogenic KS, 20 classic (27%), 14 HIV-related (19%), and 13 endemic (17%) KS.

### 3.2. Standard KS Staging According to KS Type

**Figure 1:**
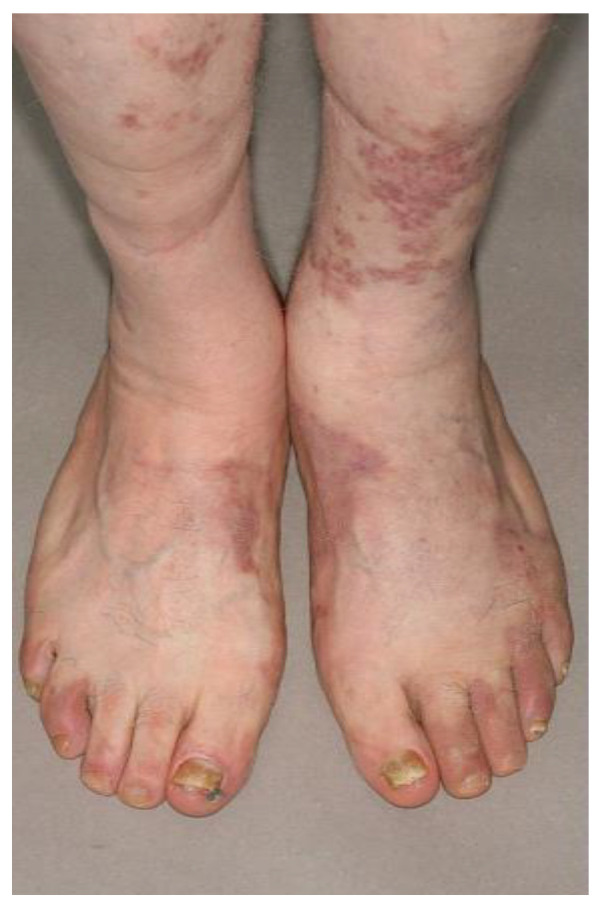
Example of a patient with classic KS with cutaneous macular extension in the lower limbs, not detected by FDG PET/CT.

With standard clinical and imaging staging, most patients had KS cutaneous involvement (67, 89%, Figure 1), and 48 patients (64%) had at least one extra-cutaneous site involved with KS. Five patients had neither cutaneous or extra-cutaneous involvement, and 70 patients had at least one site of active disease by standard staging.

Details of KS extra-cutaneous involvement by “standard staging” are provided in Table 2.

For iatrogenic KS, 50% of patients had a nodal disease, 29% had digestive involvement, and 21% had pulmonary KS (Table 2).

The most frequently involved sites in HIV-related KS were lymph nodes, mucous, and bone (43, 36, and 36%, respectively).

In classic and endemic KS, bone (20 and 31%, respectively) and lymph node involvement (20 and 31%, respectively) were the most frequent extracutaneous locations.

### 3.3. Per-Patient Analysis of Diagnostic Performance of FDG PET/CT for Staging KS

KS staging with FDG PET/CT was compared to standard staging. Sixty-one FDG PET/CT examinations (81%) were classified as positive for KS, whereas fourteen were considered negative (19%). For detecting KS involvement (cutaneous and extra-cutaneous lesions) on a per-patient basis, the sensitivity was 85%, specificity 57%, and DA 83%. When considering only patients with whole-body FDG PET/CT (*N* = 52), the sensitivity increased to 91%, with a specificity of 60% and DA of 88% (Table 3).

Forty-five (60%) FDG PET/CT examinations detected KS extra-cutaneous extension, resulting in a sensitivity of 81%, a specificity of 75%, and a DA of 84% (Table 3).

### 3.4. Overall DA on a Per-Lesion Basis

For each patient, nine sites were analyzed. Thus, for cutaneous and extra-cutaneous lesions, 675 sites were assessed in 75 patients (Table 4). FDG PET/CT yielded a sensitivity of 71%, a specificity of 98%, and a DA of 92%. When considering only the extra-cutaneous lesions, 600 sites were assessed, with diagnostic performances of sensitivity 69%, specificity 98%, PPV 83%, NPV 95%, and DA 94%. For patients with a whole-body examination (*N* = 52, 468 cutaneous sites and extra-cutaneous localizations), diagnostic performances were slightly increased with a sensitivity of 80%, specificity of 98%, and DA of 94%. For extra-cutaneous sites, 416 sites were assessed, yielding a sensitivity of 74%, specificity of 98%, and DA of 95%.

### 3.5. DA of FDG PET/CT for Cutaneous Lesions

In the whole patient population, the sensitivity of FDG PET/CT for the diagnostic of KS cutaneous involvement was 73% and the specificity 100%. More than half of FNs did not have a whole-body acquisition, and only three had non-infiltrated cutaneous lesions. Among the 52 patients (69%) who had a whole-body acquisition, sensitivity of FDG PET/CT increased to 88% (Table 5).

### 3.6. Per Site Analysis of FDG PET/CT DA for Extra-Cutaneous Lesions

The distribution of extra-cutaneous KS locations on the FDG PET/CT was lymph nodes (35, 47%), bone (14, 19%), lung (7, 9%), digestive tract (5, 7%), muscle (4, 5%), liver (3, 4%), ENT area (2, 3%), pancreas (2, 3%), and adrenal gland (1, 1%). None of these were diagnosed with mucosal (oral or genital) involvement. For each site diagnosed with KS extension, the DA of FDG PET/CT is shown in Table 5. The intensity of the FDG uptake was highly variable among KS localizations and from one patient to another (with SUVmax ranging from 1.2 to 26.7) (Suppl. Table S1).

**Figure 2:**
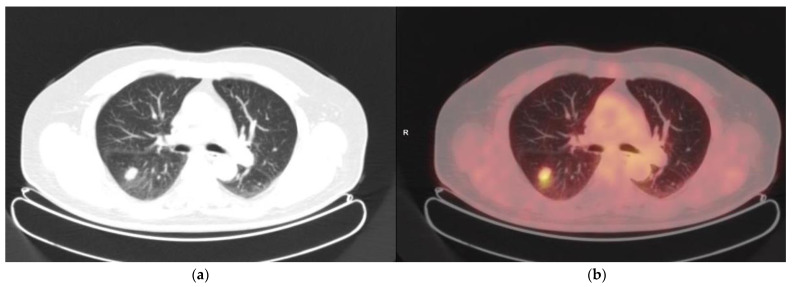
Classic KS. Hypermetabolic (SUVmax = 5) pulmonary nodule in inferior right lobe, confirmed by histology. (**a**) CT axial slice; (**b**) Fused PET/CT axial slice.

We correctly identified pulmonary KS in seven patients and missed one pulmonary involvement corresponding to non-hypermetabolic micronodules. The sensitivity was 87% and specificity 100% (Figure 2).

**Figure 3:**
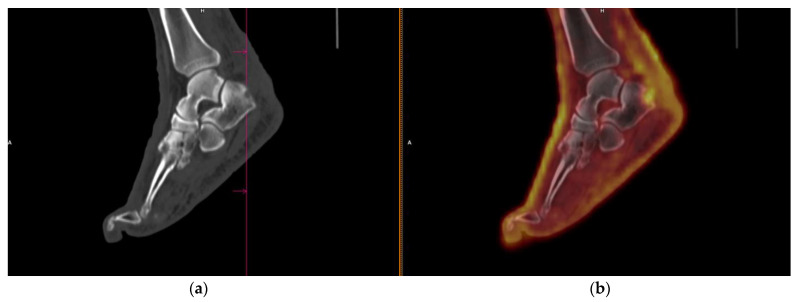
Classic KS. FDG PET/CT identifying hypermetabolic (SUV_max_ = 4.7) involvement of calcaneum contiguous to cutaneous and subcutaneous lesions. (**a**) Sagittal CT slice; (**b**) Sagittal-fused PET/CT slice.

The sensitivity was 87%, and specificity was 98% for detecting KS extension in bone. The bone lesions were lytic and mostly developed from a contiguous extension of skin or muscle lesions (Figure 3). The two FN correspond to non-hypermetabolic lytic lesions (evolutive by standard staging) in the base of a metatarsal and femur, respectively. There was one FP that corresponded to a patient with hypermetabolic extensive cutaneous, subcutaneous, and muscular infiltration in the foot, with an FDG uptake extending into the bone; however, bone involvement was not confirmed by MRI.

**Figure 4:**
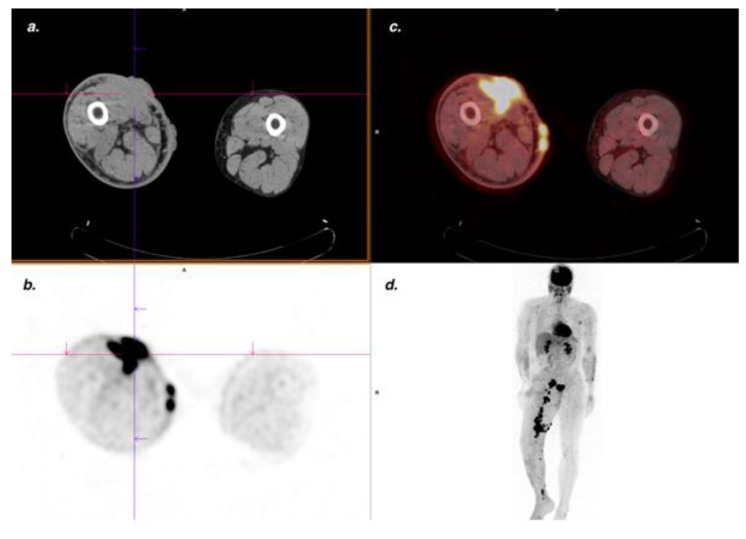

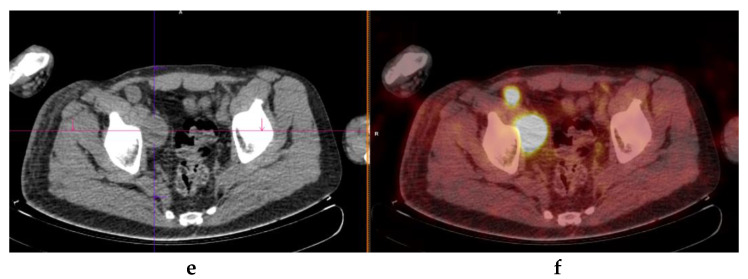
Classic KS. An FDG PET/CT showed a large hypermetabolic cutaneous lesion extending to adjacent muscles (SUV_max_ 18.6) confirmed by MRI, pathological analysis, and associated with lymph node involvement. (**a**) CT transaxial slice; (**b**) PET transaxial slice; (**c**) Fused PET/CT transaxial slice; (**d**) MIP PET image; (**e**) CT transaxial slice: right iliac enlarged lymph nodes; (**f**) Intensely hypermetabolic lymph nodes in fused PET/CT transaxial slice.

**Figure 5:**
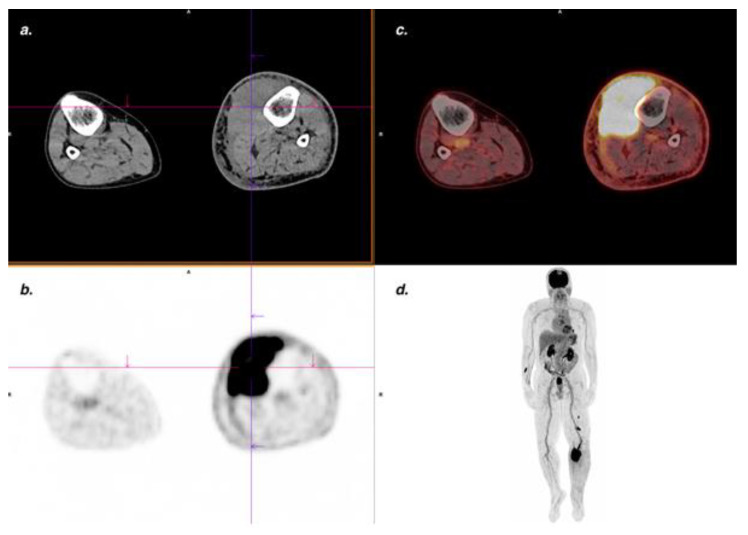
Endemic KS. Suspected clinical recurrence with increased lymphedema of left lower limb. The FDG PET/CT showed a voluminous, intensely hypermetabolic (SUV_max_ 26.7) intramuscular lesion confirmed by MRI and pathological analysis. (**a**) CT transaxial slice; (**b**) PET transaxial slice; (**c**) Fused PET/CT transaxial slice; (**d**) Maximum intensity projection (MIP) PET images.

FDG PET/CT performed well for the diagnosis of muscular KS, with four TP, no FN nor FP, yielding a sensitivity and specificity of 100%. Lesions could arise from underlying skin lesions (Figure 4) or be stand-alone lesions (Figure 5).

For nodal involvement, the sensitivity was 100%, and specificity was 85% (Figure 4). FP cases included one case of granulomatosis, one case of Hodgkin lymphoma, and five non-specific nodal uptakes.

On the contrary, FDG PET/CT failed to diagnose any mucosal involvement. Most of the mucosal location was the buccal cavity (especially the palate). Out of eight patients, one had gingival and labial lesions besides palate lesions, and one patient had a penile lesion. Digestive involvement was often missed (sensitivity 17%). The TP digestive lesions were one gastric and one colic location. FN lesions corresponded to eight gastric lesions, one colic lesion, and one patient with both gastric and colic lesions. Digestive lesions were not detected with PET mostly because of their sub-mucosal location, surrounding physiological FDG uptake, and sometimes small size.

FDG PET/CT correctly identified one pancreatic and one adrenal lesion (along with one FP pancreatic lesion and no FP adrenal lesion).

### 3.7. FDG PET/CT Results According to KS Type

We analyzed diagnostic performances by type of KS (Supplementary Table S2). FDG PET/CT performed especially well for patients with endemic KS, identifying bone, lymph nodes, and muscle involvement. In HIV-related KS, it mostly identified lymph nodes and bones. In iatrogenic KS patients it detected most frequently lymph nodes and lungs lesions, and in classic KS bone an lymph nodes lesions. Most of the missed cutaneous lesions were among patients with classic (eight patients, 29%) and iatrogenic KS (six patients, 30%).

## 4. Discussion

In this first series assessing the DA of FDG PET/CT for KS staging among 75 patients, FDG PET/CT correctly identified at least one KS lesion in more than 80% of patients with an evolutive disease and yielded a sensitivity on a per lesion basis of over 70% with variability between KS localizations.

Limited published data are available on the DA of FDG PET/CT in KS. Some case series suggested the efficacy of FDG PET/CT for KS staging, with FDG-avid lesions identified in the skin, lymph nodes, as well as occult visceral locations [17,19,24,25,26], and for follow-up during treatment [18,27,28]. Our study confirmed some of these preliminary data in a large population.

Firstly, the accuracy of FDG PET/CT in diagnosing the KS extension varied according to the sites involved. For cutaneous involvement, FDG PET/CT was relevant with an accuracy of 88% for patients with whole-body FDG PET/CT; however, the non-infiltrative, superficial cutaneous lesions could not be detected. Moreover, these patients did not have any visceral involvement, suggesting that FDG PET/CT should not be recommended for patients with only mild cutaneous lesions or no symptoms of visceral involvement in classic/endemic KS. On the contrary, for patients with an evolutive disease or an epidemic or iatrogenic KS, FDG PET/CT, including lower limbs, may be useful in identifying extra-cutaneous lesions and assessing the cutaneous extension of KS. Moreover, if systemic treatment is required, a whole-body FDG PET/CT may provide a quantitative assessment of skin involvement, which could help standardize the assessment of disease activity and clinical response in KS, and thus contribute to assess and compare KS treatments [29].

The sensitivity in detecting lymph nodes extension was excellent, with a lower specificity, due to unspecific uptake or other underlying conditions. Thus FDG PET/CT may help diagnose some diseases occurring in immunocompromised patients (lymphoma, Castleman disease, opportunistic infections, etc.) [30,31]. In our study, five patients developed a lymphoma during their follow-up. Moreover, in HIV-related KS patients, pathological lymph nodes can be detected in the advanced stages of the disease or when immunity is restored under antiretroviral therapy [32,33]. Thus, a pathological examination is recommended in case of lymph node hypermetabolism, especially in HIV-related and iatrogenic KS patients [34].

Digestive involvement of KS is the most frequent and potentially lethal visceral location in immunocompromised patients. In our study, FDG PET/CT was poorly efficient in identifying mucous and digestive lesions. For mucous lesions, this may be due to artifacts in oral and genital localizations from the salivary and urinary elimination of the tracer or obliterating pathological FDG uptake [35,36]. Moreover, the limited spatial resolution of PET/CT [37,38], the small volume of KS lesions, and their submucosal locations [16] contribute to the misdiagnosis of digestive lesions. For patients at risk of KS digestive tract involvement (iatrogenic KS or uncontrolled HIV KS, or with evocative symptoms), gastrointestinal endoscopy remains necessary for complete KS staging.

On the contrary, FDG PET/CT was very effective in detecting active musculoskeletal lesions with an accuracy of 100 and 96% for muscle and bone, respectively. These lesions usually occur from extensive local KS cutaneous lesions [9]. In our study, the distribution of these lesions across the KS types was consistent with literature data; musculoskeletal extension was observed more frequently in classic/endemic KS [6]. Bone staging is crucial for managing these patients since local or systemic treatments have limited success [6]. The ability of FDG PET/CT to accurately detect musculoskeletal lesions may also be useful in assessing treatment response because CT and MRI are not always straightforward in evaluating modifications and responses in these lesions [6].

By KS type, FDG PET/CT appears best in endemic KS with few FN examinations. Cutaneous lesions were mostly missed in patients with classic or iatrogenic KS. However, it may be useful for all types of KS, provided that the FDG PET/CT limits are known.

Our study provided important results in determining the place of FDG PET/CT in KS staging; however, it had some limitations. First, heterogeneous patients with all types of KS were included. In our study, FDG PET/CT staging and standard staging correctly reflected the various clinical presentations associated with each KS type. Half of the patients were previously treated. However, FDG PET/CT showed similar diagnostic performances in this group and the treatment-naïve patients’ group. SUVmax were not statistically different (data not shown), which may be because most previously treated patients were referred for FDG PET/CT with suspicion of recurrence. The pathological documentation of all KS lesions in each patient was not always available and may limit the interpretation. However, the gold standard was derived from a multimodal evaluation in those cases, including clinical and biological features, imaging, and follow-up data. Lastly, not all patients had whole-body imaging. At the time of the first inclusions for this study, most patients underwent FDG PET/CT to detect extra-cutaneous lesions, and at the time, a true whole-body was time-consuming (about 40 min). With the evolution of technology, a whole-body FDG PET/CT should be performed for all patients referred with KS, as mentioned in the discussion above.

This study provides a rationale for adding FDG PET/CT to the staging work-up of active KS. When performed upfront, FDG PET/CT can guide further explorations (high resolution of chest CT or bone MRI). When performed after a standard work-up, it may help characterize lesions and identify occult localizations. Moreover, it can be safely performed in renal transplant patients at high risk of visceral involvement of the disease [39].

Prospective studies are warranted to validate the ability of FDG PET/CT to correctly stratify patients according to disease stage, resulting in an improved risk-adapted therapeutic strategy.

## 5. Conclusions

Whole-body FDG PET/CT is accurate in defining cutaneous and extra-cutaneous KS extension (with the exception of mucous and digestive involvement). FDG PET/CTs can be part of the initial staging for patients at high risk of visceral or musculoskeletal involvement.

## Figures and Tables

**Table 1:** Patients’ characteristics.

Characteristics	*N* = 75 Patients (%)
Time between diagnosis and FDG PET/CT (SD), years	9 (+/−5.8)
Sex ratio (years)	4.8:1 62 men (83%) 13 women (17%)
Median age at time of FDG PET/CT (SD), years	65 (±13)
Type of Kaposi sarcoma:	
Iatrogenic ◦ renal transplant recipients ◦ long term corticosteroids ◦ autologous stem cell transplant Classic HIV-related Endemic	28 (37%) ◦ 25 (89%) ◦ 2 (7%) ◦ 1 (4%) 20 (27%) 14 (19%) 13 (17%)
Referral indication for FDG PET/CT	
Initial extension assessment Follow-up Therapeutic evaluation Suspicion of recurrence	18 (24%) 26 (35%) 6 (8%) 25 (32%)
Type of FDG PET/CT acquisitions Whole-body (from vertex to toes) No whole-body	52 (69%) 23 (31%)

**Table 2:** Distribution of involved sites according to KS type (standard staging).

	Iatrogenic *N* = 28	Classic *N* = 20	HIV-Related *N* = 14	Endemic *N* = 13	Total
Skin	22 (79%)	18 (90%)	14 (100%)	13 (100%)	67 (89%)
Lymph node	14 (50%)	4 (20%)	6 (43%)	4 (31%)	28 (37%)
Bone	2 (7%)	4 (20%)	5 (36%)	4 (31%)	15 (20%)
Digestive tract	8 (29%)	2 (10%)	2 (14%)	0 (0%)	12 (16%)
Mucous	4 (14%)	1 (5%)	5 (36%)	0 (0%)	10 (13%)
Lung	6 (21%)	1 (5%)	0 (0%)	1 (8%)	8 (10%)
Muscles	0 (0%)	1 (5%)	1 (7%)	2 (15%)	3 (4%)
ENT sphere	1 (4%)	0 (0%)	2 (14%)	0 (0%)	3 (4%)
Liver	2 (7%)	0 (0%)	1 (7%)	0 (0%)	3 (4%)
Adrenal gland	0 (0%)	0 (0%)	1 (7%)	0 (0%)	1 (1%)
Pancreas	0 (0%)	0 (0%)	1 (7%)	0 (0%)	1 (1%)

ENT: ear, nose, and throat.

**Table 3:** Per patient analysis of diagnostic performance for FDG PET/CT.

	TP	FP	TN	FN	Se	Sp	PPV	NPV	DA
All sites, all patients *N* = 75	58	3	4	10	85%	57%	95%	29%	83%
Extra-cutaneous involvement, all patients *N* = 75	38	7	21	9	81%	75%	84%	70%	79%
All sites, whole-body PET *N* = 52	43	2	3	4	91%	60%	96%	43%	88%

TP = true positive; FP = false positive; TN = true negative; FN = false negative; Sen = sensitivity; Spe = specificity; DA = diagnostic accuracy; PPV = positive predictive value; NPV = negative predictive value.

**Table 4:** Per lesion analysis of diagnostic performance for FDG PET/CT.

	TP	FP	TN	FN	Se	Sp	PPV	NPV	DA
All sites, all patients *N* = 675	106	12	513	44	71%	98%	90%	92%	92%
Extra-cutaneous lesions, all patients *N* = 600	57	12	505	26	69%	98%	83%	95%	94%
All sites, Patients with whole-body PET/CT *N* = 468	81	8	359	20	80%	98%	91%	95%	94%
Extra-cutaneous lesions Patients with whole-body PET/CT *N* = 416	39	8	355	14	74%	98%	83%	96%	95%

TP = true positive; FP = false positive; TN = true negative; FN = false negative; Sen = sensitivity; Spe = specificity; DA = diagnostic accuracy; PPV = positive predictive value; NPV = negative predictive value.

**Table 5:** Per-site analysis of diagnostic accuracy for FDG PET/CT.

KS Locations	TP	FP	TN	FN	Se	Sp	PPV	NPV	DA
Skin (all patients)	49	0	8	18	73%	100%	100%	31%	76%
Skin (patients with whole-body FDG PET/CT)	42	0	4	6	88%	100%	100%	40%	88%
Mucosal	0	0	65	10	0%	100%	NA	87%	87%
Bone	13	1	59	2	87%	98%	80%	97%	96%
Lymph nodes	28	7	40	0	100%	85%	80%	100%	91%
Lungs	7	0	67	1	87%	100%	100%	98%	99%
Digestive tract	2	3	60	10	17%	95%	40%	86%	83%
Muscle	4	0	71	0	100%	100%	100%	100%	100%
ENT sphere	2	0	72	1	67%	100%	100%	99%	99%
Liver	2	1	71	1	67%	99%	67%	99%	97%

TP = true positive; FP = false positive; TN = true negative; FN = false negative; Sen = sensitivity; Spe = specificity; DA = diagnostic accuracy; PPV = positive predictive value; NPV = negative predictive value; NA: not applicable.

## Data Availability

The data that support the findings of this study are available from the corresponding author upon reasonable request.

## Supplementary Materials

The following supporting information can be downloaded at: https://www.mdpi.com/article/10.3390/cancers14092189/s1, Table S1: Semi-quantitative analysis of FDG PET findings by localizations, Table S2: Per patient diagnostic performances according to KS type.

## References

[1] Brambilla L., Tourlaki A., Genovese G. (2017). Iatrogenic Kaposi’s Sarcoma: A Retrospective Cohort Study in an Italian Tertiary Care Centre. Clin. Oncol..

[2] Schwartz R.A., Micali G., Nasca M.R., Scuderi L. (2008). Kaposi Sarcoma: A Continuing Conundrum. J. Am. Acad. Dermatol..

[3] De Lima C.T., de Araújo P.S.R., de Teixeira H.M., dos Santos J.B., da Silveira V.M. (2017). Clinical and Laboratory Characteristics, Staging, and Outcomes of Individuals with AIDS-Associated Kaposi’s Sarcoma at an University Hospital. An. Bras. Dermatol..

[4] Lee A.J., Brenner L., Mourad B., Monteiro C., Vega K.J., Munoz J.C. (2015). Gastrointestinal Kaposi’s Sarcoma: Case Report and Review of the Literature. World J. Gastrointest. Pharmacol. Ther..

[5] Sbiyaa M., El Alaoui A., El Bardai M., Mezzani A., Lahrach K., Marzouki A., Boutayeb F. (2016). L’atteinte Osseuse Dans Le Sarcome de Kaposi Classique et Agressif: À Propos d’un Cas. Pan Afr. Med. J..

[6] Caponetti G., Dezube B.J., Restrepo C.S., Pantanowitz L. (2007). Kaposi Sarcoma of the Musculoskeletal System: A Review of 66 Patients. Cancer.

[7] Mosam A., Shaik F., Uldrick T.S., Esterhuizen T., Friedland G.H., Scadden D.T., Aboobaker J., Coovadia H.M. (2012). A Randomized Controlled Trial of HAART versus HAART and Chemotherapy in Therapy-Naïve Patients with HIV-Associated Kaposi Sarcoma in South Africa. J. Acquir. Immune Defic. Syndr..

[8] Lebbé C., Legendre C., Francès C. (2008). Kaposi sarcoma in transplantation. Transplant. Rev..

[9] Restrepo C.S., Martínez S., Lemos J.A., Carrillo J.A., Lemos D.F., Ojeda P., Koshy P. (2006). Imaging Manifestations of Kaposi Sarcoma. RadioGraphics.

[10] Mansfield S.A., Stawicki S.P.A., Forbes R.C., Papadimos T.J., Lindsey D.E. (2013). Acute Upper Gastrointestinal Bleeding Secondary to Kaposi Sarcoma as Initial Presentation of HIV Infection. J. Gastrointest. Liver Dis. JGLD.

[11] Iwamasa T., Chinen K., Hirayasu T., Nakazato I., Tsuhako K., Kamadab Y., Miyamoto K. (1996). Epidemic and Non-Epidemic Kaposi’s Sarcoma: Diagnosis, Staging and Treatment. Crit. Rev. Oncol. Hematol..

[12] Delyon J., Bizot A., Battistella M., Madelaine I., Vercellino L., Lebbé C. (2018). PD-1 Blockade with Nivolumab in Endemic Kaposi Sarcoma. Ann. Oncol..

[13] Nasti G., Tirelli U. (2005). Highly Active Antiretroviral Therapy in AIDS-Associated Kaposi’s Sarcoma (KS): Implications for the Design of Therapeutic Trials in Patients with Advanced Symptomatic KS. J. Clin. Oncol. Off. J. Am. Soc. Clin. Oncol..

[14] Lebbe C., Garbe C., Stratigos A.J., Harwood C., Peris K., Marmol V.D., Malvehy J., Zalaudek I., Hoeller C., Dummer R. (2019). Diagnosis and Treatment of Kaposi’s Sarcoma: European Consensus-Based Interdisciplinary Guideline (EDF/EADO/EORTC). Eur. J. Cancer Oxf. Engl..

[15] O’Mahony D., Gandjbakhche A.H., Hassan M., Vogel A., Yarchoan R. (2008). Imaging Techniques for Kaposi Sarcoma (KS). J. HIV Ther..

[16] Nagata N., Shimbo T., Yazaki H., Asayama N., Akiyama J., Teruya K., Igari T., Ohmagari N., Oka S., Uemura N. (2012). Predictive Clinical Factors in the Diagnosis of Gastrointestinal Kaposi’s Sarcoma and Its Endoscopic Severity. PLoS ONE.

[17] Cengiz A., Şavk E., Tataroğlu C., Yürekli Y. (2016). 18F-Fluorodeoxyglucose Positron Emission Tomography/Computed Tomography Imaging in a Patient with HIV (-) Kaposi Sarcoma. Mol. Imaging Radionucl. Ther..

[18] Morooka M., Ito K., Kubota K., Minamimoto R., Shida Y., Hasuo K., Ito T., Tasato D., Honda H., Teruya K. (2010). Whole-Body 18F-Fluorodeoxyglucose Positron Emission Tomography/Computed Tomography Images before and after Chemotherapy for Kaposi Sarcoma and Highly Active Antiretrovirus Therapy. Jpn. J. Radiol..

[19] Sager S., Engin B., Kutlubay Z., Asa S., Sager S.G., Gucluer B., Kanmaz B. (2013). PET/CT Imaging of HIV-Negative Kaposi’s Sarcoma. Ir. J. Med. Sci..

[20] Tas F., Yegen G., Keskin S., Gozubuyukoglu N. (2012). Classic Kaposi′s Sarcoma with Colonic Involvement: A Rare Presentation with Successful Treatment with Oral Etoposide. J. Cancer Res. Ther..

[21] Bleibtreu A., Mahida B., Bouscarat F., Rioux C. (2016). Asymmetric Relapse of an HIV-Associated Kaposi Sarcoma. Eur. J. Nucl. Med. Mol. Imaging.

[22] Yin L., Lin Z., Meng Z. (2021). 18F-FDG PET/CT Findings in an HIV-Infected Patient with Systemic Kaposi Sarcoma. Pol. Arch. Intern. Med..

[23] Groheux D., Biard L., Lehmann-Che J., Teixeira L., Bouhidel F.A., Poirot B., Bertheau P., Merlet P., Espié M., Resche-Rigon M. (2018). Tumor Metabolism Assessed by FDG-PET/CT and Tumor Proliferation Assessed by Genomic Grade Index to Predict Response to Neoadjuvant Chemotherapy in Triple Negative Breast Cancer. Eur. J. Nucl. Med. Mol. Imaging.

[24] Van de Luijtgaarden A., van der Ven A., Leenders W., Kaal S., Flucke U., Oyen W., van der Graaf W. (2010). Imaging of HIV-Associated Kaposi Sarcoma; F-18-FDG-PET/CT and In-111-Bevacizumabscintigraphy. J. Acquir. Immune Defic. Syndr..

[25] Reuter S., Vrachimis A., Huss S., Wardelmann E., Weckesser M., Pavenstädt H. (2014). A Challenging Case of Rapid Progressive Kaposi Sarcoma After Renal Transplantation. Medicine.

[26] Morooka M., Ito K., Kubota K., Yanagisawa K., Teruya K., Hasuo K., Shida Y., Minamimoto R., Kikuchi Y., Oka S. (2011). Usefulness of F-18 FDG PET/CT in a Case of Kaposi Sarcoma with an Unexpected Bone Lesion. Clin. Nucl. Med..

[27] Mankia S.K., Miller R.F., Edwards S.G., Ramsay A., Lee S.M. (2012). Highly Active Antiretroviral Therapy Alone Is an Effective Treatment for Lymphadenopathic Kaposi Sarcoma Demonstrated by a Clinical and F-18 FDG Positron Emission Tomography/Computed Tomography Response. Clin. Oncol..

[28] Mankia S.K., Miller R.F., Edwards S.G., Ramsay A., Lee S.M. (2012). The Response of HIV-Associated Lymphadenopathic Kaposi Sarcoma to Highly Active Antiretroviral Therapy Evaluated by 18F-FDG PET/CT. Clin. Nucl. Med..

[29] Régnier-Rosencher E., Guillot B., Dupin N. (2013). Treatments for Classic Kaposi Sarcoma: A Systematic Review of the Literature. J. Am. Acad. Dermatol..

[30] Kulasegaram R., Saunders K., Bradbeer C.S., O’Doherty M. (1997). Is There a Role for Positron Emission Tomography Scanning in HIV-Positive Patients with Kaposi’s Sarcoma and Lymphadenopathy: Two Case Reports. Int. J. STD AIDS.

[31] Polizzotto M.N., Millo C., Uldrick T.S., Aleman K., Whatley M., Wyvill K.M., O’Mahony D., Marshall V., Whitby D., Maass-Moreno R. (2015). 18F-Fluorodeoxyglucose Positron Emission Tomography in Kaposi Sarcoma Herpesvirus-Associated Multicentric Castleman Disease: Correlation with Activity, Severity, Inflammatory and Virologic Parameters. J. Infect. Dis..

[32] Davison J.M., Subramaniam R.M., Surasi D.S., Cooley T., Mercier G., Peller P.J. (2011). FDG PET/CT in Patients with HIV. AJR Am. J. Roentgenol..

[33] Mortensen B.K., Nielsen S.D., Christensen C.B., Helweg-Larsen J. (2011). Immune Reconstitution Syndrome Presenting as Probable AIDS-Related Lymphoma: A Case Report. AIDS Res. Ther..

[34] Martin C., Castaigne C., Tondeur M., Flamen P., De Wit S. (2013). Role and Interpretation of Fluorodeoxyglucose-Positron Emission Tomography/Computed Tomography in HIV-Infected Patients with Fever of Unknown Origin: A Prospective Study. HIV Med..

[35] More Y., Dusing R., Counts S., Bond J., Tsue T., Girod D. (2013). Positron-Emission Tomography Pitfalls Related to Oral Prosthesis. Laryngoscope.

[36] Makis W., Hudson E.W. (2018). Unilateral Muscle Artifacts Due to Non-Compliance During Uptake Phase of 18F-FDG PET/CT in an Oncologic Patient. Mol. Imaging Radionucl. Ther..

[37] Chipiga L., Sydoff M., Zvonova I., Bernhardsson C. (2016). Investigation of partial volume effect in different PET/CT systems: A comparison of results using the Madeira Phantom and theNEMA NU-2 2001 phantom. Radiat. Prot. Dosim..

[38] Cysouw M.C.F., Kramer G.M., Hoekstra O.S., Frings V., de Langen A.J., Smit E.F., van den Eertwegh A.J.M., Oprea-Lager D.E., Boellaard R. (2016). Accuracy and Precision of Partial-Volume Correction in Oncological PET/CT Studies. J. Nucl. Med..

[39] Delyon J., Rabate C., Euvrard S., Harwood C.A., Proby C., Güleç A.T., Seçkin D., Del Marmol V., Bouwes-Bavinck J.N., Ferrándiz-Pulido C. (2019). Management of Kaposi Sarcoma after Solid Organ Transplantation: A European Retrospective Study. J. Am. Acad. Dermatol..

