# Supplementary material for: Yield of FDG PET/CT for Defining the Extent of Disease in Patients with Kaposi Sarcoma
Source: Cancers (Basel). 2022 Apr 27;14(9):2189. doi: 10.3390/cancers14092189 (PMC9102885; doi:10.3390/cancers14092189)
Supplement: Supplementary file 1 [file cancers-14-02189-s001.zip › cancers-1661023-supplementary.pdf]

Supplementary Table S1. Semi-quantitative analysis of FDG PET findings by localizations

Site of involvement	Number of sites	Median SUVmax	SUVmax range
Skin	49	4.7	1.1–25.3
Lymph nodes	35	4.8	1.9–18.7
Bone	14	4.7	2.5–14.6
Digestive tract	5	10.3	5.3–15.4
Lungs	7	6.9	1.2–11.7
Muscles	4	10.4	5.6-26.7
ENT sphere	2	10.9	8-13.8

SUVmax: maximum standardized uptake value

Supplementary Table S2. Per patient diagnostic performances according to KS type

KS type	TP	FP	TN	FN	Se	Sp	PPV	NPV	DA
Iatrogenic *N* = 28	20	1	3	4	83%	75%	95%	43%	82%
Classic *N* = 20	14	2	1	3	82%	33%	88%	25%	70%
HIV-related *N* = 14	12	0	0	2	86%	NA	100%	NA	86%
Endemic *N* = 13	12	0	0	1	92%	NA	100%	NA	92%

TP = true positive; FP = false positive; TN = true negative; FN = false negative; Sen = sensitivity ; Spe = specificity; DA = diagnostic accuracy; PPV = positive predictive value; NPV = negative predictive value; NA: not applicable.
